# Curvature-Insensitive Transparent Surface-Enhanced Raman Scattering Substrate Based on Large-Area Ag Nanoparticle-Coated Wrinkled Polystyrene/Polydimethylsiloxane Film for Reliable In Situ Detection

**DOI:** 10.3390/molecules29122946

**Published:** 2024-06-20

**Authors:** Meng Sun, Lili Huang, Hongjun Wang, Zhaoyi Zhang, Huijuan Niu, Zhenshan Yang, Hefu Li

**Affiliations:** Key Laboratory of Optical Communication Science and Technology of Shandong Province, School of Physical Science and Information Technology, Liaocheng University, Liaocheng 252000, China; sunmeng9804@163.com (M.S.); 15885296453@163.com (L.H.); acborn@163.com (H.W.); yili161@163.com (Z.Z.); supernhj@lcu.edu.cn (H.N.); yangzhenshan@lcu.edu.cn (Z.Y.)

**Keywords:** SERS, Ag NP-coated wrinkled PS/PDMS film, curvature insensitivity, quantitative detection, point-of-care testing

## Abstract

Flexible and transparent surface-enhanced Raman scattering (SERS) substrates have attracted considerable attention for their ability to enable the direct in situ detection of analytes on curved surfaces. However, the curvature of an object can impact the signal enhancement of SERS during the measurement process. Herein, we propose a simple approach for fabricating a curvature-insensitive transparent SERS substrate by depositing silver nanoparticles (Ag NPs) onto a large-area wrinkled polystyrene/polydimethylsiloxane (Ag NP@W-PS/PDMS) bilayer film. Using rhodamine 6G (R6G) as a probe molecule, the optimized Ag NP@W-PS/PDMS film demonstrates a high analytical enhancement factor (AEF) of 4.83 × 10^5^, excellent uniformity (RSD = 7.85%) and reproducibility (RSD = 3.09%), as well as superior mechanical flexibility. Additionally, in situ measurements of malachite green (MG) on objects with diverse curvatures, including fish, apple, and blueberry, are conducted using a portable Raman system, revealing a consistent SERS enhancement. Furthermore, a robust linear relationship (R^2^ ≥ 0.990) between Raman intensity and the logarithmic concentration of MG detected from these objects is achieved. These results demonstrate the tremendous potential of the developed curvature-insensitive SERS substrate as a point-of-care testing (POCT) platform for identifying analytes on irregular objects.

## 1. Introduction

Surface-enhanced Raman scattering (SERS) is a powerful non-invasive spectroscopic technique that enables the ultrasensitive identification of various analytes through their unique molecular fingerprints [1]. Due to its label-free, rapid, and non-destructive detection capabilities, SERS technology has found widespread applications in fields such as food safety [2,3], environmental monitoring [4], and biomedicine [5,6]. The significant enhancement of Raman signals in SERS primarily results from electromagnetic enhancement induced by the plasmonic coupling effect at nanogaps among metallic nanostructures, known as “hot spots” [7,8]. In order to achieve the convenient and effective trace determination of analytes, the sampling method is a crucial consideration in the design and construction of SERS substrates. Conventional rigid SERS substrates pose challenges as they resist conformal contact with curved surfaces, complicating the sample collection procedure. To address this issue, flexible SERS substrates made of materials such as polymer film [9], adhesive tape [10,11], cellulose paper [12,13], and cotton [14,15] have been proposed and developed. These flexible SERS substrates facilitate wipe sampling on curved surfaces due to their ability to make conformal contact [16]. In particular, targeted analytes on curved surfaces can be detected in situ by covering the objects with flexible and transparent SERS substrates, providing a straightforward and rapid detection strategy that aligns well with the requirements of point-of-care testing (POCT) [17,18].

Various polymer materials, such as polydimethylsiloxane (PDMS) [19,20], polymethyl methacrylate (PMMA) [21,22], and polyethylene terephthalate (PET) [23,24], are widely employed as supporting films for constructing flexible and transparent SERS substrates. Among these, PDMS is an ideal support material for constructing flexible and transparent SERS substrates due to its good flexibility and transparency, high mechanical strength, and low scattering cross-section [25]. Moreover, it allows for the facile preparation of micro/nanostructures on its surface. To achieve high sensitivity and reproducibility in in situ detection, it is beneficial to create three-dimensional (3D) structures on flexible supports. This approach increases the number of hot spots and capture sites while conforming more closely to complex surfaces [26]. However, current methods for creating these structures, such as molding and oxygen plasma treatment, are not suitable for the high-throughput production of SERS substrates in a simple and cost-effective manner [27,28,29,30,31,32,33]. Therefore, there is an urgent demand for a simple, efficient, and affordable technology that can create structured 3D features on large-scale PDMS films. Moreover, for flexible sensors to be used in daily applications, they must consistently generate a uniform signal regardless of the bending of the underlying substrates [34]. Additionally, these sensors should exhibit high signal enhancement. Despite these requirements, the challenge of fabricating a curvature-insensitive, flexible, and transparent SERS substrate that is suitable for large-scale mass production still persists.

**Figure 1:**
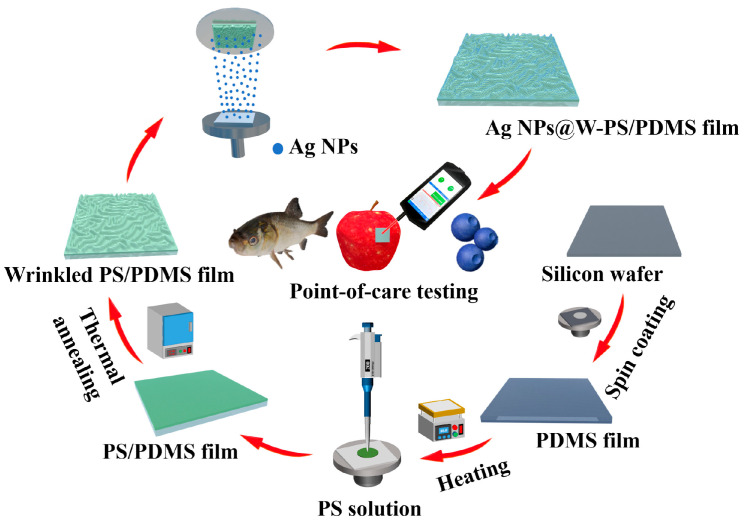
Schematic illustration of the fabrication procedure of the Ag NP@W-PS/PDMS film and its POCT analysis on objects with diverse curvatures.

Here, we fabricated a large-area 3D flexible SERS substrate consisting of a wrinkled polystyrene/polydimethylsiloxane bilayer film decorated with Ag NPs (Ag NP@W-PS/PDMS). This substrate allows for rapid and quantitative detection on diverse surfaces while maintaining high sensitivity and consistency, as illustrated in Figure 1. The fabrication process involved the use of thermal treatment to create a structured PS/PDMS bilayer film with anisotropic wrinkles. Subsequently, Ag NPs were deposited onto these wrinkles using magnetron sputtering to generate 3D hot spots. The Ag NP@W-PS/PDMS film exhibited high sensitivity and excellent uniformity and reproducibility, as demonstrated by spectral testing with rhodamine 6G (R6G). In addition, mechanical testing of the flexible SERS substrate revealed no degradation in enhancement performance even after 100 cycles of repeated deformations. Finally, the in situ detection of malachite green on various surfaces, including those with different curvatures such as fish, apple, and blueberry, showed consistent and reliable SERS signals produced by the Ag NP@W-PS/PDMS film. These results highlight the potential of this curvature-insensitive transparent Ag NP@W-PS/PDMS film for SERS-based POCT.

## 2. Results and Discussion

**Figure 2:**
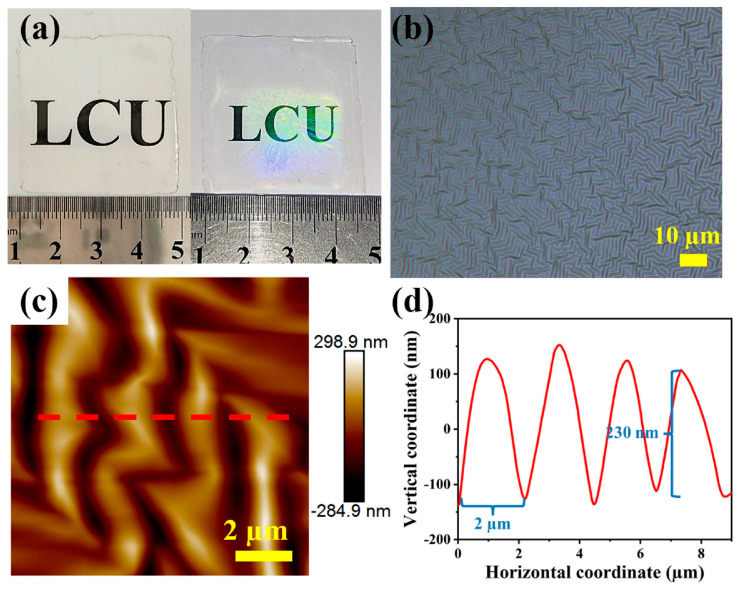
(**a**) Photographs of a flat PDMS film (left) and a PS/PDMS bilayer film after thermal treatment (right). (**b**) Optical microscope image and (**c**) AFM image of surface wrinkles on the PS/PDMS bilayer film. (**d**) The height profile of the wrinkled structure is marked with a red line in (**c**).

To achieve a large-scale pattern structure on the PDMS film, thermally induced buckling was used to generate wrinkles in the PS/PDMS bilayer system as a result of the different coefficients of thermal expansion between the two materials [35]. The photograph in Figure 2a shows a comparison between a flat PDMS film (left) and a thermally treated PS/PDMS bilayer film (right). Notably, the PS/PDMS bilayer film displays a distinct grating effect, indicating the generation of thermally induced wrinkles. Moreover, this wrinkled bilayer film maintains excellent optical transparency. Figure 2b shows the surface morphology of the prepared PS/PDMS bilayer film, revealing the presence of anisotropic wrinkle structures. The characterization by atomic force microscopy (AFM), depicted in Figure 2c, further confirms the morphology of these wrinkles. The amplitude and peak-to-peak distance of the wrinkle structures are approximately 230 nm and 2 µm, respectively (Figure 2d). With a low aspect ratio of around 0.115, these wrinkle structures offer a large surface area for the deposition of Ag NPs, and meanwhile, they improve the availability of hot spots toward target analytes.

**Figure 3:**
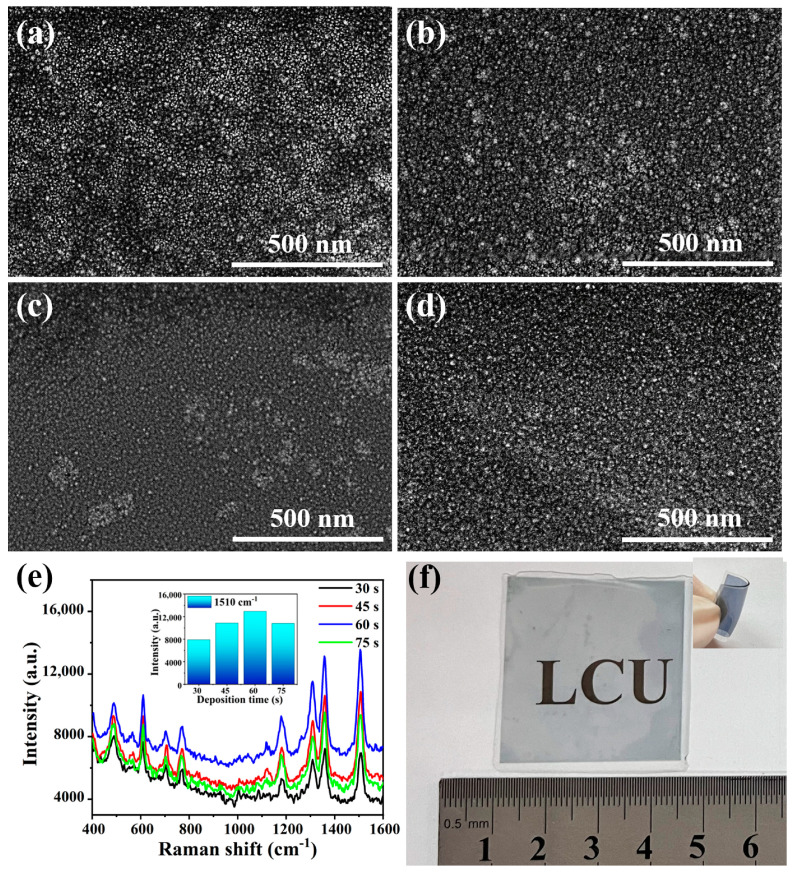
SEM images of the Ag NP@W-PS/PDMS films with (**a**) 30 s, (**b**) 45 s, (**c**) 60 s, and (**d**) 75 s Ag sputtering times. (**e**) SERS spectra of 10^−5^ M R6G collected from different Ag NP@W-PS/PDMS films. The inset shows the comparison of SERS intensity between different substrates. (**f**) Photograph of a large-area transparent Ag NP@W-PS/PDMS film on a flat paper. Inset shows a photograph of the deformed Ag NP@W-PS/PDMS film, demonstrating the substrate flexibility.

To optimize the SERS activity of the Ag NP@W-PS/PDMS films, Ag sputtering was performed with varying durations ranging from 30 to 75 s. The surface morphologies of Ag NP@W-PS/PDMS films, prepared with different sputtering times, were examined, as shown in Figure 3a–d. It was observed that the structural morphology was influenced by the Ag deposition time, resulting in different enhancement performances. The SERS spectra of R6G at a concentration of 10^−5^ M collected on these Ag NP@W-PS/PDMS films are illustrated in Figure 3e. The SERS intensity at 1510 cm^−1^ showed an increase with the extension of sputtering time from 30 to 60 s but decreased when the sputtering time was further extended to 75 s (as shown in the inset of Figure 3e). This variation in SERS activity can be attributed to the morphological changes in the Ag NPs with the sputtering time. At a shorter deposition time (30 s), small nanoparticles with large nanogaps were unable to generate effective hot spots (Figure 3a). As the Ag sputtering time increased, the distance between the Ag NPs gradually decreased, leading to the formation of more hot spots that enhanced SERS signals (Figure 3b). Particularly at a sputtering time of 60 s, a high density of ultra-small and uniform nanogaps was formed on the surface (Figure 3c), resulting in maximum enhancement. However, with further prolongation of the sputtering time (Figure 3d), some of the Ag NPs began to connect, diminishing the number of hot spots and attenuating the signal intensity. Consequently, the Ag NP@W-PS/PDMS-60 film was chosen for subsequent experiments.

Furthermore, our proposed fabrication strategy enables the construction of a large-area SERS substrate with outstanding flexibility and transparency, as illustrated in Figure 3f. The blue color observed is attributed to the localized surface plasmon effect of Ag NPs [36]. Subsequently, a detailed analysis of the surface elements of the Ag NP@W-PS/PDMS-60 film was conducted using energy-dispersive X-ray spectrometry (EDS). The results of the EDS analysis revealed that Ag, Si, O, and C elements constituted the surface composition of the Ag NP@W-PS/PDMS-60 film (Figure S1a). Moreover, EDS mapping demonstrated a uniform distribution of the Ag element on the surface of the wrinkled PS/PDMS film (Figure S1b). 

**Figure 4:**
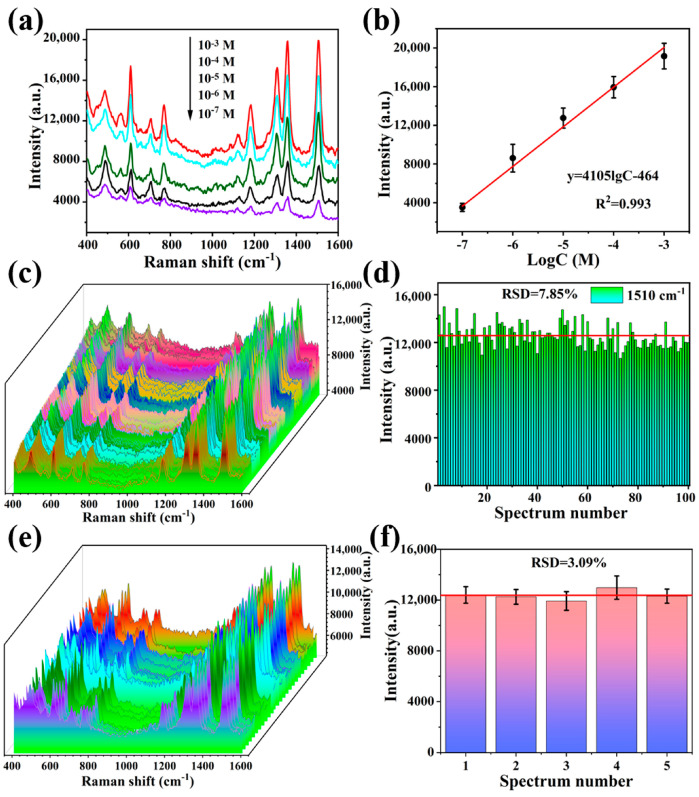
(**a**) SERS spectra of R6G with different concentrations obtained from the Ag NP@W-PS/PDMS-60 film. (**b**) Linear correlation of peak intensity at 1510 cm^−1^ with the logarithmic concentration of R6G. (**c**) SERS spectra of R6G (10^−5^ M) collected from 100 random sites. (**d**) The histogram of SERS intensity of R6G at 1510 cm^−1^ at the 100 sites. The red line represents the average intensity. (**e**) SERS spectra of R6G (10^−5^ M) collected from five different Ag NP@W-PS/PDMS-60 films. (**f**) The histogram of SERS intensity of R6G at 1510 cm^−1^ from 5 different substrates. The red line represents the average intensity.

To evaluate the SERS sensitivity of the Ag NP@W-PS/PDMS-60 film, SERS spectra of R6G at various concentrations (10^−3^ to 10^−7^ M) were measured. As depicted in Figure 4a, the signal intensity decreased with decreasing R6G concentration, and even at a low concentration of 10^−7^ M, the characteristic peaks of R6G remained clear and distinguishable. Additionally, the calibration curve correlating the Raman intensity at 1510 cm^−1^ with the logarithm of R6G concentration in the range of 10^−3^–10^−7^ M exhibited a good linear relationship with an R^2^ value of 0.993 (Figure 4b). This suggests that the Ag NP@W-PS/PDMS-60 film possesses a quantitative analysis capability. Furthermore, the analytical enhancement factor (AEF) of the Ag NP@W-PS/PDMS-60 film was calculated using the following formula [37]: AEF = (I_SERS_/C_SERS_)/(I_RS_/C_RS_). Here, I_SERS_ and I_RS_ represent signal intensities of the analyte under SERS and non-SERS conditions, respectively, while C_SERS_ and C_RS_ denote analyte concentrations used for SERS and non-SERS measurements. For this calculation, the signal intensity of R6G at 1510 cm^−1^ was selected, with I_SERS_ and I_RS_ being 4141 and 857, respectively. Additionally, C_SERS_ and C_RS_ were 10^−7^ M and 10^−2^ M, respectively (Figure S2). Consequently, the AEF of the SERS substrate was calculated to be 4.83 × 10^5^.

Uniformity and reproducibility are critical factors for ensuring reliable detection in SERS. To evaluate the substrate uniformity, SERS spectra of R6G (10^−5^ M) were obtained from 100 randomly chosen locations within the Ag NP@W-PS/PDMS-60 film. The measurements were taken from areas at the center, middle, and edge of the substrate. The SERS spectra exhibited nearly identical peak shapes and intensities, as shown in Figure 4c. The relative standard deviation (RSD) of peak intensity at 1510 cm^−1^ was calculated to be 7.85% (Figure 4d), indicating consistent and reliable detection signals from the Ag NP@W-PS/PDMS-60 film. For batch-to-batch reproducibility, SERS measurements were conducted in five different batches, with five R6G SERS signals collected from each substrate (Figure 4e). The RSD value of peak intensity at 1510 cm^−1^ was determined to be 3.09%, as shown in Figure 4f. These results demonstrate the excellent uniformity and reproducibility of the obtained flexible SERS substrate. Additionally, Table S1 presents comparisons of analytical enhancement factor, uniformity, and reproducibility between the Ag NP@W-PS/PDMS substrate and other flexible SERS substrates. The substrate may have exhibited lower enhancement factors compared to some other flexible SERS substrates [25,29,31,38,39,40,41,42,43,44]. This could be attributed to variations in substrate surface roughness and plasmonic material structure or composition, as well as differences in experimental conditions. However, its advantage lies in its ability to provide the consistent and reliable in situ detection of surface analytes across various curvature profiles, making it valuable for versatile and accurate surface analysis applications.

**Figure 5:**
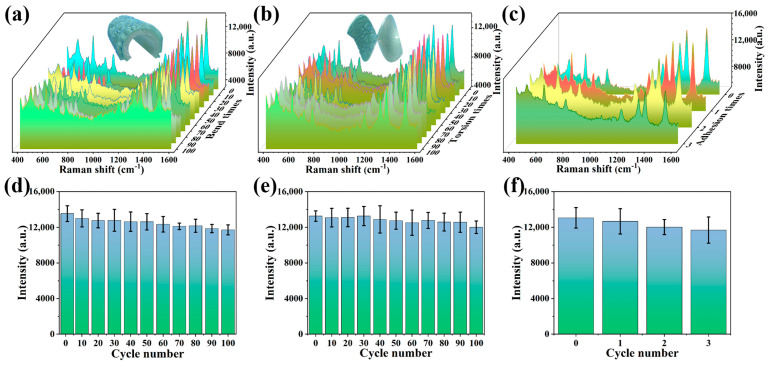
SERS spectra of R6G collected from Ag NP@W-PS/PDMS-60 film in (**a**) bending, (**b**) twisting, and (**c**) adhesion test. SERS intensities of R6G at 1510 cm^−1^ recorded from Ag NP@W-PS/PDMS-60 film in (**d**) bending, (**e**) twisting, and (**f**) adhesion test.

Mechanical stability is also a crucial consideration for real-world applications of flexible SERS substrates. The robustness of the Ag NP@W-PS/PDMS-60 film was evaluated by measuring the SERS spectra of R6G (10^−5^ M) every 10 instances of continuous bending in half or twisting to ~180° (Figure 5a,b). Remarkably, there was only a small alteration in SERS intensity at the peak of 1510 cm^−1^ during 100 cycles of bending or torsion, as illustrated in Figure 5d,e. This demonstrates the excellent mechanical durability of the Ag NP@W-PS/PDMS-60 film. The robust adhesion between the Ag NPs and the wrinkled PS/PDMS film likely contributes to this resilience. To confirm the strong adhesion, a cohesiveness test was conducted by affixing strong tape to the substrate surface and then peeling it off. Figure 5c displays the SERS spectra of R6G collected from the Ag NP@W-PS/PDMS-60 film during three cycles of the “paste and peel off” operation. Across these cycles, no significant change in SERS intensity at peak 1510 cm^−1^ was observed (Figure 5f), indicating robust adhesion between the Ag NPs and the wrinkled PS/PDMS film. These results collectively demonstrate that the Ag NP@W-PS/PDMS-60 film maintains structural integrity and stability under repeated mechanical deformations, facilitating the reusability of the substrate.

**Figure 6:**
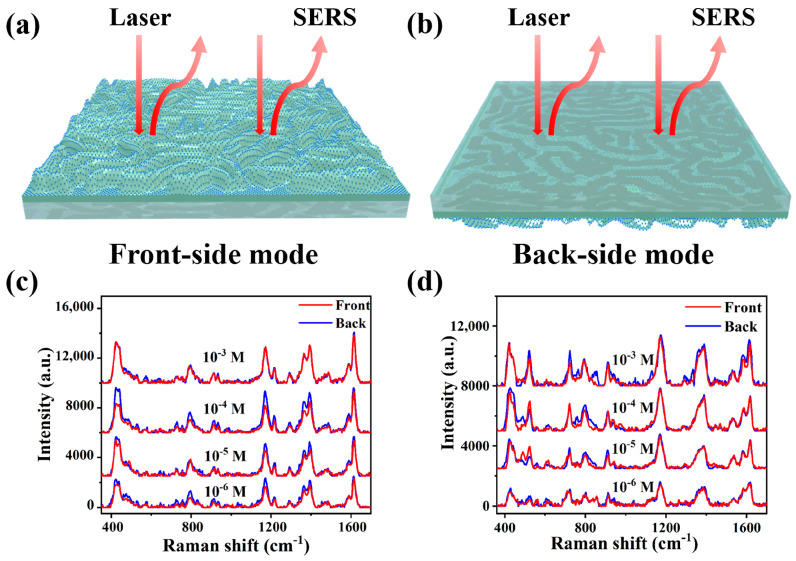
Schematic illustration of SERS measurements under (**a**) front-side and (**b**) back-side excitation mode. Comparison of SERS spectra of (**c**) MG and (**d**) CV with concentrations ranging from 10^−3^ to 10^−6^ M obtained from both sides of the Ag NP@W-PS/PDMS-60 film.

To enable direct in situ detection, it is essential to ensure the excitation and collection of the SERS signal from the back side of the SERS substrate. The bidirectional activation characteristic of Ag NP@W-PS/PDMS-60 film was evaluated using two different measurement modes, the front-side mode and back-side mode, as depicted in Figure 6a,b. The SERS spectra of malachite green (MG) and crystal violet (CV) at concentrations ranging from 10^−3^ to 10^−6^ M were collected on the transparent SERS substrate under both modes, as shown in Figure 6c,d. Remarkably, the SERS spectra obtained under both measurement modes were consistent, showing no apparent weakening of the SERS signal intensity. This result demonstrates the suitability of the prepared Ag NP@W-PS/PDMS-60 film as a flexible and transparent SERS substrate for in situ detection.

**Figure 7:**
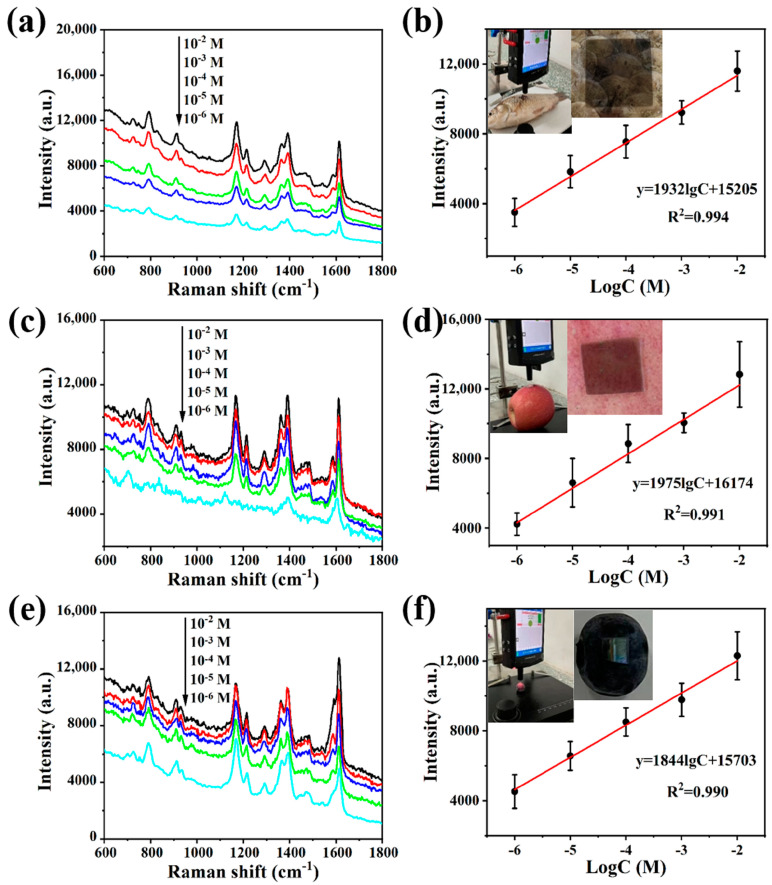
SERS spectra of MG with different concentrations on the surfaces of (**a**) fish and (**c**) apple and (**e**) blueberry acquired by in situ detection using Ag NP@W-PS/PDMS-60 film. The plot of peak intensity at 1616 cm^−1^ versus the logarithm of MG concentration collected from the surfaces of (**b**) fish and (**d**) apple and (**f**) blueberry. The insets in (**b**,**d**,**f**) are photographs showing portable in situ detection measurements.

Given its excellent mechanical stability and superior bidirectional activation characteristic, the Ag NP@W-PS/PDMS-60 film is well suited for the in situ detection of analytes on complex surfaces. To assess its applicability on real samples with different curvatures, MG was chosen as a model pollutant. For SERS measurements, 20 μL of MG solution at various concentrations was dropped to the surfaces of real objects (fish, apple, and blueberry) with diverse curvatures. Following natural drying, the Ag NP@W-PS/PDMS-60 film was applied to the contaminated surfaces, and in situ SERS measurements were conducted using a portable Raman spectrometer (insets of Figure 7). Figure 7a,c,e illustrate the SERS spectra of MG at different concentrations (10^−2^–10^−6^ M) on the surface of fish, apple, and blueberry, respectively. Even at a low concentration of 10^−6^ M, the SERS signals of MG on these real samples were clearly identifiable. The comparison of SERS signals collected from different objects reveals that the Ag NP@W-PS/PDMS-60 film exhibited slight variations in curvature-induced fluctuations in signal intensity. This suggests that the film can function as a curvature-insensitive transparent SERS substrate for in situ detection on curved surfaces, maintaining detection performance regardless of the object’s curvature. Figure 7b,d,f show the corresponding correlation between the SERS intensity at the peak of 1616 cm^−1^ and the logarithm of MG concentration on the surfaces of the fish, apple, and blueberry. A good linear relationship was observed for all three surfaces, with R^2^ values of 0.994, 0.991, and 0.990, respectively. These results demonstrate that the Ag NP@W-PS/PDMS-60 film, with its excellent surface adaptability, can be easily employed in daily life for reliable in situ detection when combined with a portable Raman spectrometer.

The consistent signal enhancement observed on actual objects with different curvatures can be attributed to several key factors. Firstly, the thin and flexible nature of the Ag NP@W-PS/PDMS-60 film allows it to conform perfectly to the surfaces of objects, as is evident in the insets of Figure 7b,d,f. Secondly, the low aspect ratio of the wrinkled structures in the film provides effective and accessible hot spots for target analytes. Thirdly, the disordered nature of the wrinkled structures helps to average the density and strength of hot spots across the substrate. Lastly, the distribution of hot spots remains stable even under large strain conditions, thanks to the mechanical stability of the PS/PDMS film and the structural stability of the Ag NPs. Consequently, the flexible Ag NP@W-PS/PDMS-60 film can be used as a versatile SERS substrate with broad applicability, owing to its mechanical suitability and consistent signal enhancement characteristics.

## 3. Materials and Methods

### 3.1. Materials

The silver target was purchased from Zhongnuo Advanced Material Technology Co., Ltd. (Beijing, China). Silicon wafer and polydimethylsiloxane (PDMS) were bought from Hangzhou Jingbo Technology Co., Ltd. (Hangzhou, China). Rhodamine 6G (R6G) was obtained from Sigma Aldrich. Polystyrene (PS), crystal violet (CV), and malachite green (MG) were obtained from Aladdin Reagent Co., Ltd. (Shanghai, China). Deionized (DI) water was used to prepare aqueous solutions.

### 3.2. Preparation of Ag NP@W-PS/PDMS Film

The flat PDMS film was initially prepared by mixing the pre-polymer and its curing agent (10:1, *w*/*w*). After mechanical stirring for 20 min and subsequent vacuum degassing for 30 min, the mixture was spin-coated onto the surface of a cleaned silicon wafer (4 cm × 4 cm) at 600 rpm for 8 s and cured at 80 °C for 12 h. The obtained PDMS film was then exposed to oxygen plasma (PDC-002, Harrick Plasma, Ithaca, NY, USA) at 30 W for 30 s to change the surface hydrophilicity to help the spin-coating of PS solution. Next, 200 μL of PS solution in toluene (1 wt%) was spin-coated onto the surface of the plasma-treated PDMS film at 2500 rpm for 60 s. After that, the PS-coated PDMS film was placed in a vacuum oven for 2 h to remove residual organic solvent in the PS film. Subsequently, the sample was annealed at 200 °C for 1 h and taken out of the oven and cooled at room temperature in air. Due to the difference in thermal expansion coefficients between PS and PDMS, compressive stress was generated at the interface of PS/PDMS bilayer film, inducing disordered surface patterns. Finally, the wrinkled PS/PDMS film was decorated with Ag NPs using the magnetron sputtering technique. During the deposition of Ag NPs, the sputtering power, vacuum, and working pressure were 20 W, 5.0 × 10^−4^ Pa, and 0.8 Pa, respectively. The deposition time varied between 30 s, 45 s, 60 s, and 75 s.

### 3.3. Characterization

Optical microscopy (Zeiss-Imager A2m, Oberkochen, Germany) was used to characterize the prepared PS/PDMS bilayer film. The as-prepared SERS substrates were characterized by atomic force microscopy (Bruker Dimension Icon SPM, Bruker Corp., Billerica, MA, USA) and a field-emission scanning electron microscope (FESEM, Regulus 8100, HITACHI, Tokyo, Japan). The SERS spectra were collected at room temperature using a smartphone-based Raman analyzer (ATR6500, Optosky photoelectric Co., Ltd., Xiamen, China) with a laser of 785 nm (20 mW) and acquisition time of 10 s.

### 3.4. SERS Measurements

To evaluate the SERS performance of the Ag NP@W-PS/PDMS substrate, rhodamine 6G (R6G), malachite green (MG), and crystal violet (CV) were selected as probe molecules. These molecules were dissolved in deionized water to prepare standard analyte solutions with varying concentrations. Subsequently, 10 µL of each analyte solution was dropped onto the surface of the flexible SERS substrate and allowed to naturally dry at room temperature. SERS measurements were then conducted using a handheld Raman spectrometer. For in situ detection measurements, 20 µL of MG solution with different concentrations was dropped on the surface of fish, apple, and blueberry. After drying, the Ag NP@W-PS/PDMS flexible SERS substrate was wrapped around the surfaces of samples, with Ag NPs facing them, and SERS measurements were implemented by using a handheld Raman spectrometer. 

## 4. Conclusions

A flexible and transparent SERS substrate was successfully fabricated using a simple manufacturing process involving the generation of thermal-induced wrinkles in a PS/PDMS bilayer film and the deposition of Ag NPs through magnetron sputtering. The resulting Ag NP@W-PS/PDMS film exhibited high SERS sensitivity and homogeneity due to the presence of high-density and uniform hot spots on the wrinkled structures. The strong adhension between the Ag NPs and the PS/PDMS film enhanced the mechanical stability of the flexible SERS substrate, while also maintaining exceptional optical transparency and enabling rapid and in situ detection on curved surfaces. Moreover, the flexible Ag NP@W-PS/PDMS SERS substrate demonstrated insensitivity to variations in curvature, as evidenced by the reproducible signal intensities and excellent quantitative analysis results obtained during in situ detection measurements of MG on fish, apple, and blueberry surfaces. The proposed flexible Ag NP@W-PS/PDMS film offers a range of benefits, including ease of fabrication, high sensitivity with outstanding reproducibility, excellent mechanical stability, and insensitivity to changes in curvature. As a result, it holds great potential for applications in SERS-based POCT.

## Data Availability

Data are contained within the article.

## Supplementary Materials

The following supporting information can be downloaded at https://www.mdpi.com/article/10.3390/molecules29122946/s1: Figure S1. (a) EDS spectrum and (b) EDS mapping of Ag NP@W-PS/PDMS-60 flexible SERS substrate. Figure S2. SERS spectra of R6G (10^−7^ M) collected from the Ag NP@W-PS/PDMS-60 film and Raman spectra of R6G (10^−2^ M) collected from the pure PDMS film. Table S1. Comparisons between some recent reported flexible SERS substrates.
