# Supplementary material for: Curvature-Insensitive Transparent Surface-Enhanced Raman Scattering Substrate Based on Large-Area Ag Nanoparticle-Coated Wrinkled Polystyrene/Polydimethylsiloxane Film for Reliable In Situ Detection
Source: Molecules. 2024 Jun 20;29(12):2946. doi: 10.3390/molecules29122946 (PMC11206736; doi:10.3390/molecules29122946)
Supplement: Supplementary file 1 [file molecules-29-02946-s001.zip › molecules-2963870-supplementary.pdf]

Supplementary Materials

Curvature-Insensitive Transparent Surface-Enhanced Raman Scattering Substrate Based on Large-Area Ag Nanoparticle-Coated Wrinkled Polystyrene/Polydimethylsiloxane Film for Reliable In Situ Detection

Figure S1

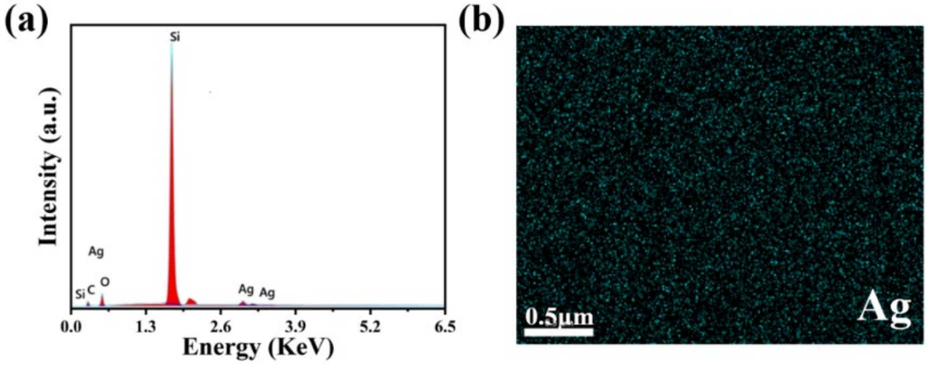

Figure S1. (**a**) EDS spectrum and (**b**) EDS mapping of Ag NP@W-PS/PDMS-60 flexible SERS substrate.

Figure S2

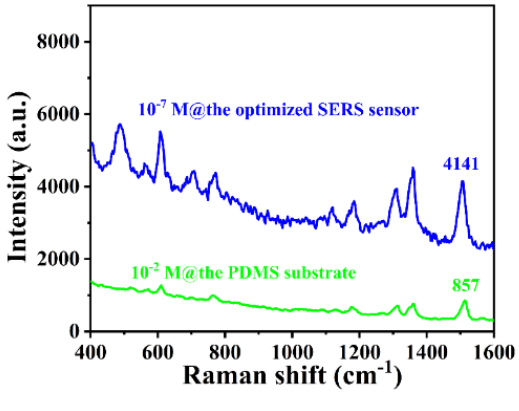

Figure S2. SERS spectra of R6G (10^{-7} M) collected from the Ag NP@W-PS/PDMS-60 film and Raman spectra of R6G (10^{-2} M) collected from the pure PDMS film.

Table S1. Comparisons between some recent reported flexible SERS substrates.

SERS substrate	Probe analyte	AEF	Uniformity	Reproducibility	Reference
Flexible pyramid array	R6G	2.1×10^{7}	6.3%	4.6%	25
Open nanocavity-assisted Ag@PDMS	R6G	$\sim10^{12}$	6.86%		29
MLA/WS/AgNPs 3D-SERS	R6G	4.35×10^{7}	6.12%		31
AgNCs@SiO2/PMHS modified paper substrate	R6G	6.55×10^{6}	6.05%	9.22%	38
triangular Au/Ag nanoparticle arrays	R6G	2.13×10^{8}	2.3%	6.3%	39
Ag NPs@W-PDMS film	R6G	1.19×10^{5}	6.27%	7.3%	40
cotton SERS substrates	PATP	5.18×10^{5}	8.15%		41
Biomimetic flexible SERS substrates	R6G	4.2×10^{5}	7.3%	2.8%	42
Au NBPs@Ag/PDMS film	CV	1.5×10^{7}	12.7%		43
spiky gold nanostars	R6G	6.2×10^{8}	5.4%		44
Ag NP-coated wrinkled PS/PDMS film	R6G	4.83×10^{5}	7.85%	3.09%	this work
